# Climatological hazard indicators for a robust and integrated energy infrastructure in Austria

**DOI:** 10.1038/s41597-025-06121-2

**Published:** 2025-11-24

**Authors:** Philipp Maier, Lukas Liebmann, Kristofer Hasel, Romana Berg, Fabian Lehner, Marianne Bügelmayer-Blaschek, Herbert Formayer, Demet Suna

**Affiliations:** 1 Institute of Meteorology and Climatology, BOKU University, Gregor-Mendel-Straße 33, 1180 Vienna, Austria; 2 Center for Energy, AIT Austrian Institute of Technology, Giefinggasse 4, 1210 Vienna, Austria

**Keywords:** Climate-change impacts, Energy infrastructure, Renewable energy

## Abstract

The energy sector faces constant challenges due to the anticipated green transition and the underlaying change of climate. To be able to assess potential risks during the transition towards renewable energy sources, we present ROBINE-AT, an impact-oriented climatological dataset for Austria, consisting of 41 hazard maps for the Global Warming Levels 1.0 °C (corresponding to 2001–2020), 2.0 °C, 3.0 °C and 4.0 °C. The maps cover potential hazards for Austria’s energy system due to various impacts, including heat and cold stress, wind, extreme precipitation, floods, droughts, humidity, lightning strikes, and wildfires. The data is based on six localized and bias-adjusted climate projections of the CMIP5 and CMIP6 generation. With a diverse set of indicators and a high spatial resolution of 1 km, ROBINE-AT provides an innovative blueprint for assessing climate hazards, targeting regions with complex mountainous terrain such as Austria. Additionally, insights into climate impacts on the energy sector are provided, enabling tailored risk assessments by combining the hazards with custom exposure and vulnerability data.

## Background & Summary

Climate change influences multiple levels of society, including its energy systems and infrastructure^1^. The range of impacts - particularly on the electricity infrastructure - are diverse and especially dependent on the chosen energy sources. They encompass, among others, reduction of thermal power plant efficiency due to rising temperatures^2^, outages of transmission lines due to wildfires^3^ and failure of wind turbine power converters due to increased humidity^4^. In addition, the European Union’s anticipated transition to a climate neutral energy system requires strong renewable-based electrification, which inherently is more affected by weather and climate^5,6^.

**Fig. 1:**
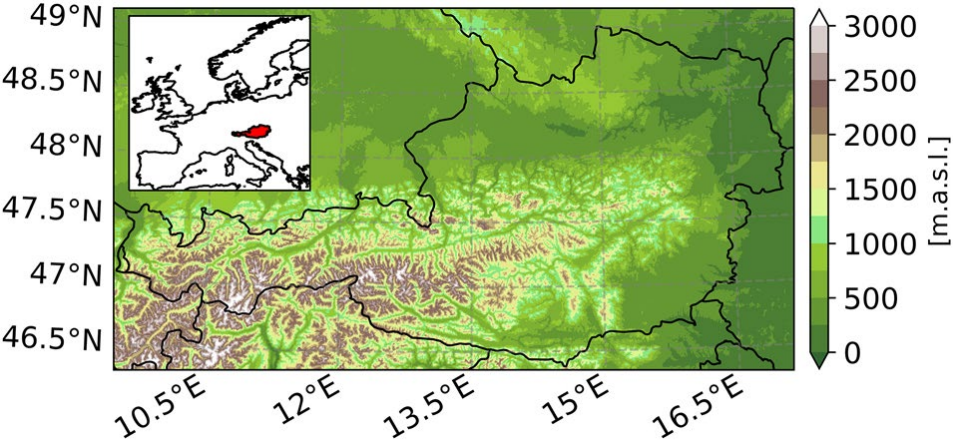
Topography of Austria and surroundings in meters above sea level, obtained from^98,99^. The inner panel shows the location of Austria in Europe.

While qualitative analysis on the various impacts of climate change on the energy system exists^1^ and relevant climate indicators have been derived in other geographical areas^7^, no data set so far focuses on providing climatological data fulfilling the requirements for a multi-impact analysis on energy systems in regions with complex, mountainous orography. Within the research project “ROBINE”, experts from the fields of energy modelling, climate impact studies and urban climate modelling therefore generated a collection of climate hazard maps for a ROBust and INtegrated Energy infrastructure in AusTria (ROBINE-AT) specifically targeting this research gap. This work is novel in its inclusion of a broad range of relevant indicators, considering various weather extremes to comprehensively assess the potential impacts on energy systems. The data set covers Austria with a spatial resolution of 1 km as a suitable example of a mountainous region. Figure 1 shows Austria’s geographical location in Europe and its topography in meters above sea level and highlights its complex orography, especially in the alpine region of Western Austria. The alpine west is contrasted by the comparatively dry and flat east, where Vienna is located. Furthermore, Austria presents a compelling study region as the main ridge of the Alps acts as a significant meteorological divide, separating the country’s climate into a predominantly Atlantic-influenced north and a Mediterranean-influenced south, whereas northeastern regions also exhibit continental climatic influences^8^. By adopting the Global Warming Level (GWL) approach^9^ - defined as 20-year periods, during which the global mean surface temperature exceeds the pre-industrial average (185–1900) by a specified amount - we provide climate projection data independent of emission scenarios for the GWLs 2.0 °C, 3.0 °C and 4.0 °C as well as observations for the historical period of 2001–2020, which corresponds to GWL-1.0 °C. The GWL approach shifts the view from emission scenarios to an impact-oriented approach. Since climate projections from different generations yield comparable climate change signal patterns for the same GWL^10^, our approach can be replicated using alternative climate models that provide the most plausible results for other geographical regions. In combination with vulnerability and exposure information as the framework of the Intergovernmental Panel on Climate Change (IPCC) suggests^11,12^, these hazard maps serve as basis to assess specific use-case dependent risks for energy systems and infrastructure.

The core information about this data set is compiled in Tables 1 and 2. Table 1 describes the considered climate hazard (first column), the impact indicators available as hazard maps (second column) and their meteorological definition (third column). Examples on how the various energy system components are potentially impacted by the respective hazard are visible in Table 2, separated into the three main pillars of the energy sector: demand (second column), supply (third column) and infrastructure (fourth column). The symbols in the columns indicate whether the displayed hazards have a positive (increasing) effect (+), a negative (decreasing) effect (–), or a bidirectional effect (±). The assessment of the impacts of weather events on energy systems follows a structured approach that integrates data collection, literature review, stakeholder engagement, and indicator development (see section 2). On the supply side, changes in weather conditions influence output from wind, solar, and hydro^13,14^, while changes in temperatures influence thermal power plant efficiency^15^. Additionally, hydropower generation is at risk due to shifts in precipitation patterns and water availability^16–18^. In terms of energy demand, temperature variations significantly influence heating and cooling needs, while seasonal and extreme weather fluctuations impact the electricity consumption patterns and accordingly load profiles^13^. Further, infrastructure resilience is endangered as extreme weather events such as floods, storms and heatwaves pose risks to electricity system and gas network components^17,19–21^. The presented indicators quantify the frequency, intensity, and duration of relevant meteorological events and their potential consequences. Temperature-related indicators (heat and cold hazards) include weather events such as heatwaves, cold spells, and degree days (Heating Degree Days - HDD, Cooling Degree Days - CDD). Precipitation, flood and drought hazard indicators like the Standardized Precipitation Evapotranspiration Index (SPEI) cover the effect of heavy respectively low precipitation on energy system components. Wind-related indicators, including maximum wind speeds and calm days, are crucial for assessing impacts on wind energy generation. Additionally, wildfires, humid days and lightning strikes are considered in both tables.

**Table 1:** Hazard indicators presented in ROBINE-AT.

Hazard	Indicator name	Definition
Heat	desert days	annual average number of days with ${T}_{\max }\,\ge$ 35
	average longest period of desert days	annual average longest period of consecutive days with ${T}_{\max }\,\ge$ 35 °C
	days in heat waves	annual number of days in heat waves as defined by Kysely *et al*.^47^
	average longest period of heat wave days	annual average longest period of heat wave days as defined by Kysely *et al*.^47^
	average maximum temperature	average annual maximum temperature
	absolute maximum temperature	absolute maximum temperature occurring in the GWL period
	tropical nights	annual average number of days with ${T}_{\min }\,\ge$ 20 °C
	average longest period of tropical nights	annual average longest period of consecutive days with ${T}_{\min }\,\ge$ 20 °C
	cooling degree days	annual average number of days with *T*_mean_ ≥ 22.5 °C, as defined by Pezzutto *et al*.^61^
	average longest period of cooling degree days	annual average longest period of consecutive days with *T*_mean_ ≥ 22.5 °C, as defined by Pezzutto *et al*.^61^
	average maximum soil temperature	annual average maximum soil temperature in 1m-depth (see section 2 for description)
	warm soil days	annual average number of days with *T*_soil_ ≥ 20 °C in 1 m depth (see section 2 for description)
Cold	frost days	annual average number of days with ${T}_{\min } <$ 0 °C
	average longest period of frost days	annual average longest period of consecutive days with ${T}_{\min }\, <$ 0 °C
	ice days	annual average number of days with ${T}_{\max } <$ 0 °C
	average longest period of ice days	annual average longest period of consecutive days with ${T}_{\max } <$ 0 °C
	extreme ice days	annual average number of days with ${T}_{\max }\, <$ 0 °C and ${T}_{\min }\, <$ −7 °C (regional definition^64^)
	average longest period of extreme ice days	annual average longest period of consecutive days with ${T}_{\max }\, <$ 0 °C and ${T}_{\min }\, <$ −7 °C (regional definition^64^)
	ice throw days	annual average number of days with ${T}_{\min }\, <$ −20 °C at 150 m (turbine) height (see section 2 for description)
	heating degree days	annual average number of days with *T*_mean_ < 12 °C (regional definition^64^)
	heating degree sum	summed temperature difference of all days with *T*_mean_ < 12 °C to room temperature (20 °C, regional definition^64^)
Precipitation	average maximum daily precipitation	annual average maximum daily precipitation
	average maximum three-day precipitation	annual average maximum three-day precipitation
	wet snowfall days	annual average number of days where (*T*_850hPa_ + *T*_500hPa_)/2 ∈ [−15 °C, − 10 °C] and pr ≥ 10 mm (see section 2 for description)
	snowfall days	annual average number of days where *T*_mean_ < 0.5 °C and the two-day pr ≥10 mm
Flooding	fluvial flood days	annual average number of days where precipitation enables fluvial floods, as defined by Kaitna *et al*.^49^
	debris flow days	annual average number of days where precipitation enables debris flow, as defined by Kaitna^49^
Wind	storm days	annual average number of days with maximum gusts gusts ≥90 km/h
	strong storm days	annual average number of days with maximum gusts gusts ≥120 km/h
	extreme wind gust speed	95^th^ percentile of annual NUTS-averaged storm gust (see section 2 for description)
	calm days	annual average number of days with wspd < 2.5 m/s in 150 m (turbine) height (see section 2 for description)
	average longest period of calm days	annual average longest period of consecutive days with wspd < 2.5 m/s in 150 m (turbine) height (see section 2 for description)
Fire	very high fire danger days	annual average number of days with fire weather index FWI ≥38 (very high risk^65^), as defined by van Wagner^50^
	extreme fire danger days	annual average number of days with FWI ≥50 (extreme risk^65^), as defined by van Wagner^50^
Drought	years with one-month drought	number of years including at least one month within May and November with SPEI < −2^48^
	years with three-month drought	number of years including a three-month period within May and November with SPEI < −2^48^
	years with extreme low flow	number of years including a three-month period within July and November with SPEI < −3^48^, skipping potential snow melt in May and June
Humidity	humid days	annual average number of days with dew point *T*_d_ ≥ 20 °C
	average longest period of humid days	annual average longest period of consecutive days with dew point *T*_d_ ≥ 20 °C
Lightning	thunderstorm days	annual average number of days with lightning probability *p* ≥ 0.5, as defined by Laimighofer *et al*.^42^
	lightning-induced fire danger days	annual average number of days with FWI ≥ 38, as defined by van Wagner^50^ and lightning probability *p* ≥ 0.5, as defined by Laimighofer *et al*.^42^

**Table 2:** Impacted energy system components.

Hazard	Demand	Supply	Infrastructure
Heat	cooling (+)^13^	hydropower (−)^13^	power lines (−)^13^
	heating (−)^13^	thermal power plants (−)^13,15^	power substations (−)^66^
		photovoltaics (PV, ± )^13^	battery charging (−)^67^
		biomass growth (±)^68^	gas compressors (−)^21^
		battery storage (−)^67^	gas pipelines (−)^69^
Cold	cooling (−)^13^	hydropower (−)^70–72^	power lines (−)^13^
	heating (+)^13^	thermal power plants (+)^73^ (−)^74^	battery charging (−)^75–77^
		PV (+)^78,79^ (−)^79,80^	gas pipelines (−)^74,81^
		biomass growth (+)^68^	
		near-ground geothermal energy (−)^82^	
		battery storage (−)^79^	
		pumped hydro (−)^71,72,83^	
		wind (−)^52,84,85^	
Precipitation	electricity (−)^20^	hydropower (±)^17,18^	electricity transmission and distribution networks (−)^17,19,20^
		PV (−)^86^	
Flooding	electricity (−)^20^	hydropower (−)^17,18^	electricity transmission and distribution networks (−)^17,19,20^
		PV (−)^14^	
		biomass growth (±)^14^	
Wind	electricity (−)^15^	PV (−)^14^	electricity transmission and distribution networks (−)^15^
		wind (±)^14^	
		biomass growth (±)^14^	
Fire	electricity (−)^15^	biomass growth (−)^15^	electricity transmission and distribution networks (−)^15^
Drought	electricity (−)^15^	hydropower (−)^15^	electricity transmission and distribution networks (−)^15^
		biomass growth (−)^15^	
Humidity		PV (−)^87^	electricity transmission and distribution networks (−)^88^
		wind (−)^89^	power substations (−)^90^
Lightning		PV (−)^91^	electricity transmission and distribution networks (−)^92,93^
		wind (−)^94,95^	substations and transformers^96^
		thermal power plants (−)^97^	

By integrating climate impact indicators, literature research, and stakeholder perspectives, this methodology provides a systematic approach to understand and address climate risks in the energy sector. The structured identification of relevant indicators supports decision-makers in developing climate adaptation strategies, enhancing system resilience, and ensuring sustainable energy system planning. This comprehensive approach ensures that energy systems can withstand the challenges posed by climate change and continue to operate reliably in the face of increasing climate variability in the coming decades^22^.

**Fig. 2:**
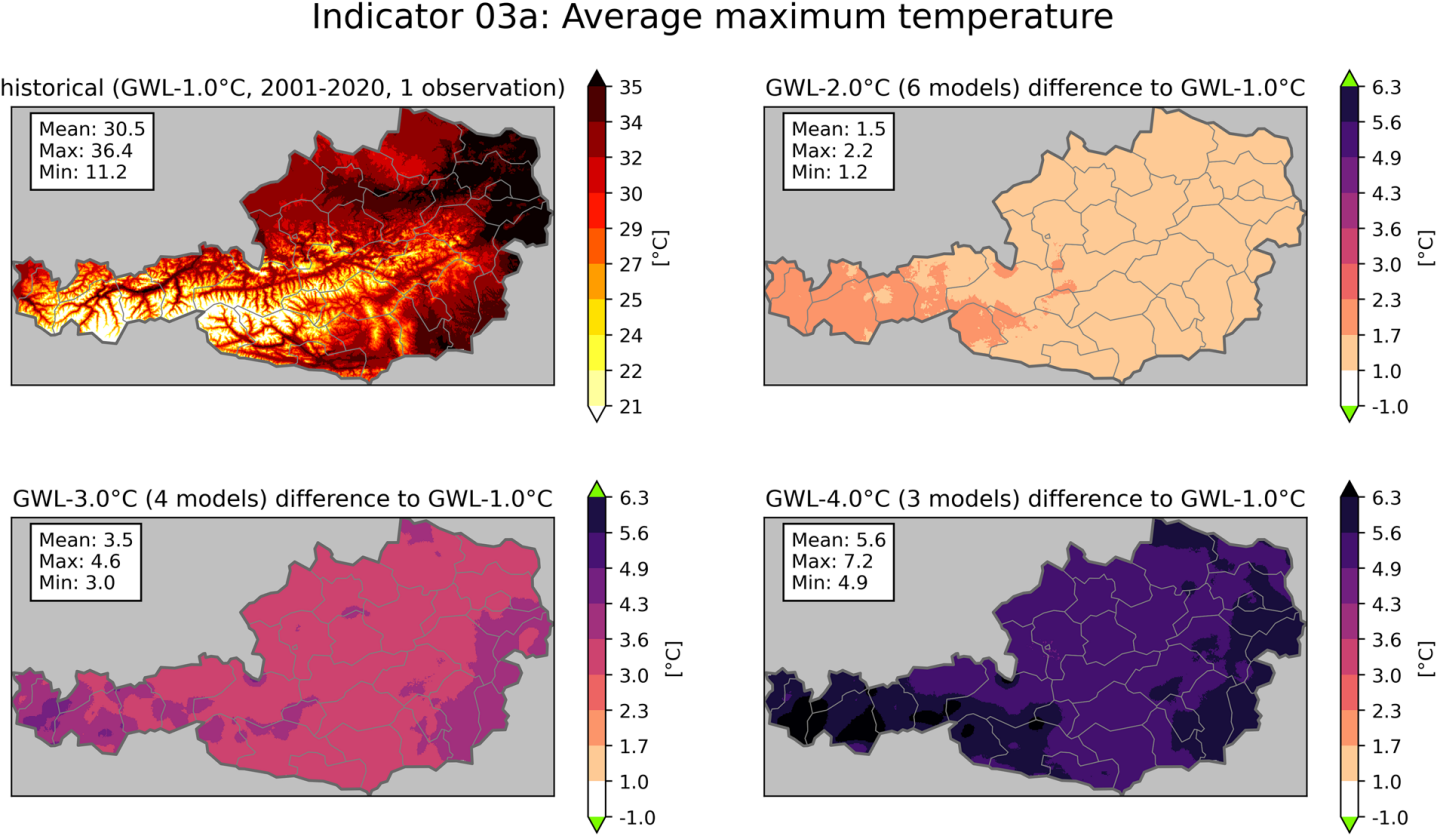
Indicator 03a - average maximum temperature in °C. The figure shows the historical period (top left) and the averaged climate change signal for three GWLs. Mean, maximum and minimum values are displayed in the text boxes. Green colors in the colorbar indicate, that no value is present for this class. The numbers in brackets show, how many climate projections were used to generate the average climate change signal of the GWL.

All presented indicators and their corresponding maps are openly available for download^23^. Table 4 shows the units of the individual indicators as well as their assigned number used in file names. Further, some map examples are presented here: As a proxy for potential heat hazards, Fig. 2 shows the effect of global warming on the annual maximum temperature in Austria, averaged over the 20-year GWL periods. While the historical values (top left), corresponding to 2001–2020 and therefore GWL-1.0 °C, show a heavy dependency on elevation with temperatures ranging from 11.2 °C in the highest alpine regions to 36.4 °C in Austria’s low-lying east, the climate change signals (remaining panels) do not show strong elevation-dependency. The increase in Austria’s spatial average maximum temperature exceeds the expected GWL temperature changes in all warming scenarios, with the largest discrepancy at GWL-4.0 °C, where the spatial average of the annual maximum temperature rises by 5.6 °C. This emphasizes that temperature extremes intensify more significantly than average temperatures^24^.

**Fig. 3:**
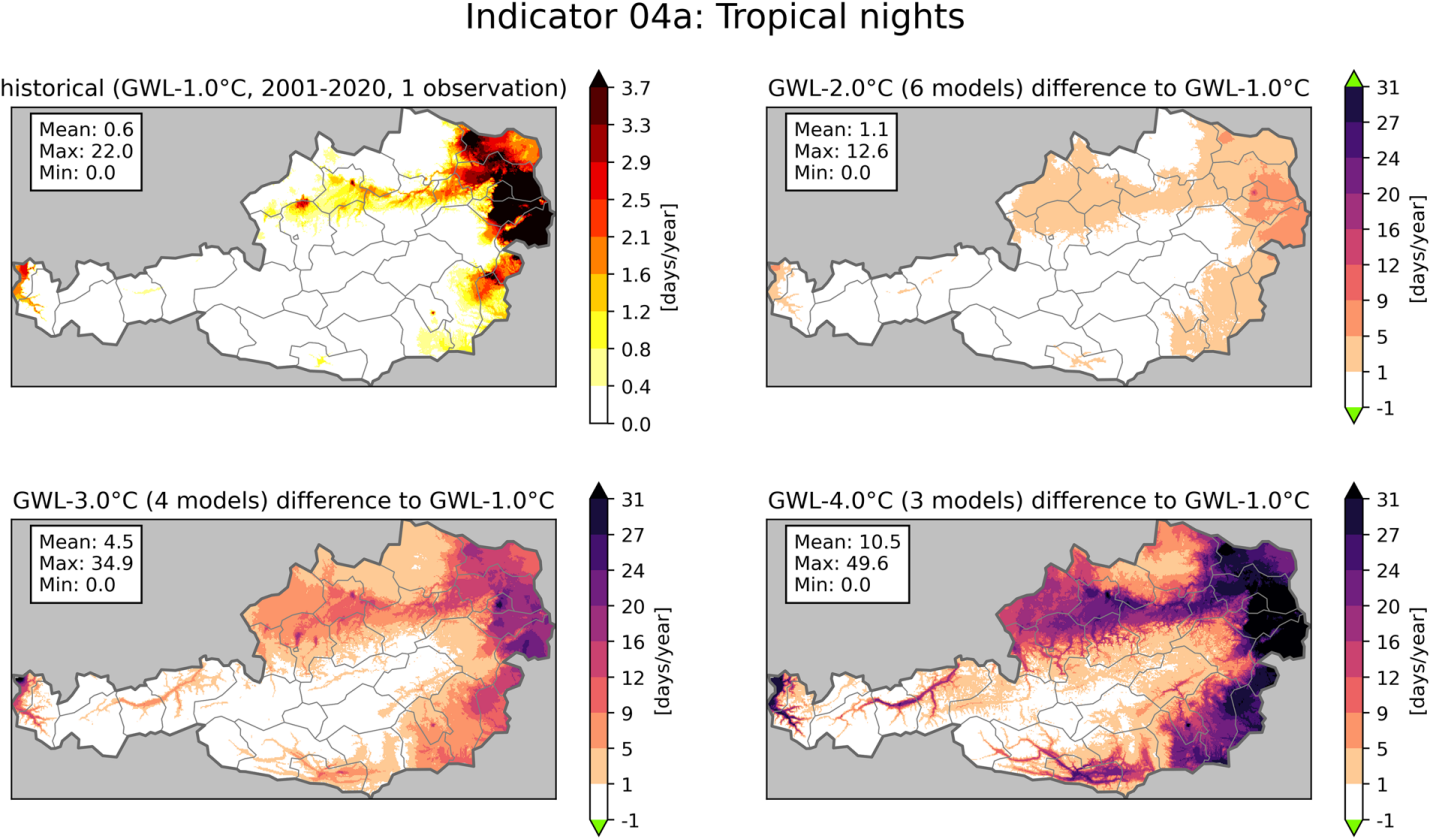
Indicator 04a - days with tropical nights in days/year. The figure shows the historical period (top left) and the averaged climate change signal for three GWLs. Mean, maximum and minimum values are displayed in the text boxes. Green colors in the colorbar indicate, that no value is present for this class. The numbers in brackets show, how many climate projections were used to generate the average climate change signal of the GWL.

Figure 3 presents another heat-related hazard: the annual average number of tropical nights (defined as days with ${T}_{\min }\,\ge 20{,}^{\circ }$C), expressed in days per year. The panels follow the same structure as in Fig. 2. In the historical data (top left), tropical nights are mainly present in Austria’s low-lying east and in the Rhine valley. Starting from GWL-2.0 °C, tropical nights emerge in the valleys of Drava and Inn as well as in Austria’s south. A strong positive trend is visible, with an average increase of 10.5 days per year for whole Austria in GWL-4.0 °C and the strongest increase in the city center of Vienna with 49.6 days per year. Furthermore, despite being a temperature-dependent indicator like Fig. 2, applying a threshold produces a climate change signal, which shows elevation dependency. This behaviour is not present in Fig. 2 and therefore highlights the importance of showing a large range of different indicators and thresholds.

**Fig. 4:**
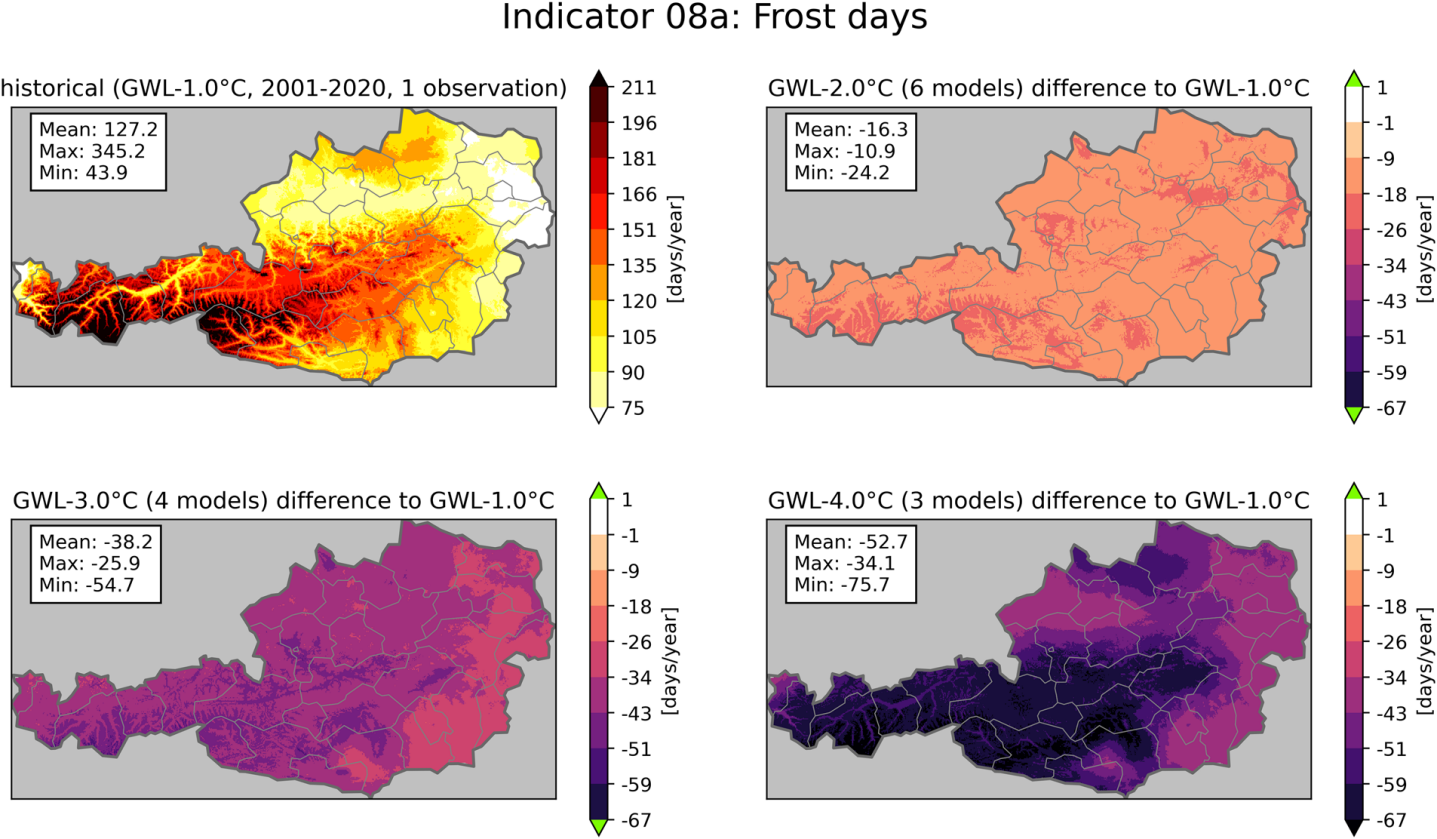
Indicator 08a - frost days in days/year. The figure shows the historical period (top left) and the averaged climate change signal for three GWLs. Mean, maximum and minimum values are displayed in the text boxes. Green colors in the colorbar indicate, that no value is present for this class. The numbers in brackets show, how many climate projections were used to generate the average climate change signal of the GWL.

Figure 4 shows the frost days (defined as ${T}_{\min } <$ 0 °C) in days per year as an example for a cold hazard. As expected, with increasing global temperatures, this indicator shows the highest values in the historical period of 2001–2020 (top left), whereas the three GWLs in the remaining panels show decreasing frost days. The trend severity is heavily dependent on elevation with the strongest decrease in high-alpine areas. Similar plots for all 41 available indicators are also provided for download in addition to the data^23^.

## Methods

### Assessing the effects on energy infrastructure

Information on the climate impacts on the energy infrastructure is gathered from existing scientific literature, technical reports, and industry insights to understand known, expected, and potential climate change impacts on energy infrastructure, system operation, and planning. Literature review sources include scientific studies on climate impacts on energy infrastructure^25,26^ and case studies of past extreme weather events affecting energy systems^27,28^. In parallel, stakeholder engagement is conducted through consultations with grid operators, energy utilities and other interest groups. Workshops, interviews and feedback sessions with industry representatives provide valuable insights into impacted energy system components, adaptation strategies and risk mitigation measures. By combining findings from literature and stakeholder perspectives, a well-rounded understanding of climate hazards and risks to energy systems is established. Both assessments involve identifying relevant climate impact indicators associated with extreme weather events, which are subsequently constructed with the methods described below to assess their potential effects on different components of the energy system.

### Base climate data

**Fig. 5:**
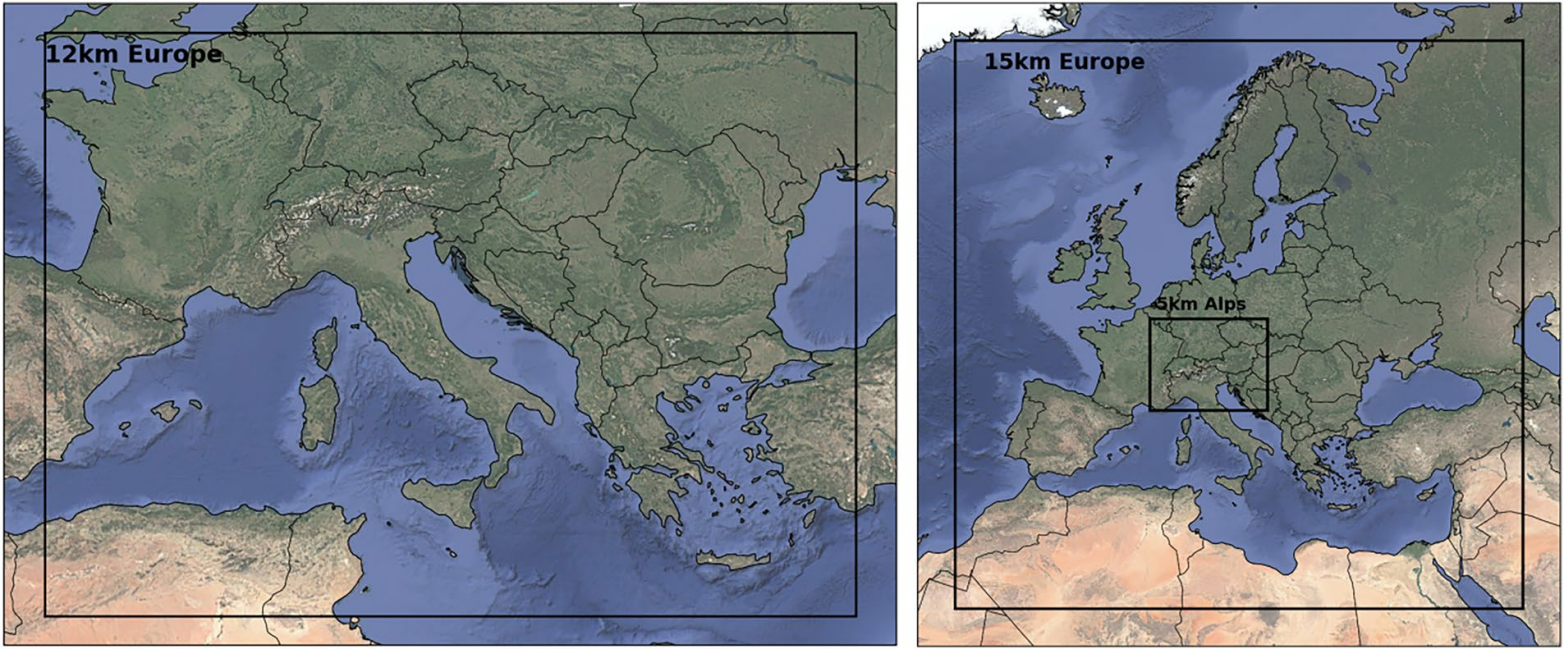
CCLM (left) and WRF (right) simulation domains. CCLM: Central European domain with a 12 km spatial resolution. WRF: European domain with a 15 km spatial resolution; nested domains at 5 km resolution.

The historical data^29^ used for this data set was derived from data sets and station data of the Austrian weather service GeoSphere Austria^30,31^ and expanded with required meteorological variables on a daily basis with a resolution of 250 m. For the hazard maps, the data is reprojected on a 1 km grid using patch interpolation^32^ to be able to accurately represent climatic conditions in complex orography. The future projections used for this data encompass three EURO-CORDEX models^33^ of the fifth generation of the Coupled Model Intercomparison Project (CMIP5)^34^ and three General Circulation Models (GCMs) of the CMIP6 generation^35^, whereas the latter are dynamically downscaled and publicly available^36^. For this dynamical downscaling of the CMIP6 models, which are also part of the EURO-CORDEX initiative^37^, two Regional Climate Models (RCMs) were employed, each initialized with outputs from the selected CMIP6 GCMs. The COSMO-CLM (CCLM,version 4.8-19)^38,39^ model was used to downscale EC-EARTH3-Veg, while the Weather Research & Forecasting model (WRF, version 4.3.3)^40^ was applied to downscale MPI-ESM1-2-HR. The respective domains for dynamical downscaling are shown in Fig. 5. The domain differences arise from resolution variations: WRF simulations were conducted at a 15 km and a 5 km spatial resolution respectively, while CCLM simulations used a 12 km resolution for its Central European domain. All simulations provide hourly outputs and extend until the year 2100.

The name of the future projections, including the GCM, emission scenario, ensemble run and RCMs as well as their GWL periods, in which the global mean temperature surpasses a certain threshold for a 20-year average, are listed in Table 3. After regridding the models to the same grid as the historical data, the data is bias-adjusted using the historical data^29^ from the period 1991–2020 as ground truth and quantile mapping as suggested by^41^. Used variables include the daily mean, minimum and maximum temperature, dew point temperature, precipitation, potential evapotranspiration, 10m-wind and 10m-gust speed and lightning probability^42^ as well as noon values for temperature, relative humidity and wind speed. Further, the raw data of ERA5^43^ and the future projections are used for temperatures at the pressure levels 500 hPa and 850 hPa. These meteorological variables within the GWL periods serve as basis for this data set and are further compiled to climate indicators as suggested by Table 1.

**Table 3:** Models and time periods of GWLs for each model used in this study.

Model	GWL-1.0 °C	GWL-2.0 °C	GWL-3.0 °C	GWL-4.0 °C
Historical observations	2001–2020	—	—	—
ICHEC-EC-EARTH_rcp85_r12i1p1_SMHI-RCA4	1994–2013	2025–2044	2051–2070	2073–2092
MPI-M-MPI-ESM-LR_rcp45_r1i1p1_CLMcom-CCLM4-8-17	1993–2012	2035–2054	—	—
MPI-M-MPI-ESM-LR_rcp85_r1i1p1_CLMcom-CCLM4-8-17	1993–2012	2028–2047	2052–2071	2072–2091
EC-Earth3-Veg_SSP126_r1i1p1f1_COSMO-CLMv5-12	1984–2003*	2020–2039	—	—
EC-Earth3-Veg_SSP585_r1i1p1f1_COSMO-CLMv5-12	1984–2003*	2018–2037	2041–2060	2058–2077
MPI-ESM1-2-HR_SSP585_r1i1p1f1_WRFv4-3-3	2003–2022	2040–2059	2063–2082^†^	—

^*^Due to the availability of bias adjusted data only from 1991 onwards, the seven years before were constructed by determining and chaining together climatologically similar periods in the SSP1-2.6 run on a seasonal basis.

^†^Original period would be 2064–2083, but data was missing for 2083.

### Indicator calculation

Most climate indicators presented in this data sets are value over respectively under threshold indicators^44^, whereas the used thresholds are visible in Table 1. These thresholds are commonly used in regional practical applications and science communication to the general public and stakeholders by. e.g. the Austrian national weather service^45^ and in case of regionalised definitions, are grounded in standards to be applicable to Austrian infrastructure and building types^46^. Other indicators, namely days in heat waves^47^, the Standardized Precipitation Evapotranspiration Index (SPEI)^48^, thresholds for fluvial and debris flows^49^, fire weather index (FWI)^50^ and lightning probability^42^ follow existing definitions and methodologies, which are referenced accordingly in the tables. Indicators, where new methods are used, are described here:

- Soil temperature: Soil temperature in one meter depth is calculated by determining a linear regression using existing station data of the 50-day average of the 2m-air temperature and the daily 1m-soil temperature, provided by GeoSphere Austria^51^. Daily data was available from 13 stations, with an average record length of 13.6 years. This regression yields for the soil temperature on day d
  1 $${T}_{soil,d}=0.777\cdot \frac{1}{50}\mathop{\sum }\limits_{i=d-49}^{d}{T}_{air,i}+3.751,$$
  with an r^2^-score of 0.947 and is subsequently applied on the gridded data sets.
- Ice throw days: Ice throw days are calculated by adjusting the minimum 2m-temperature to 150 m using a lapse rate of *Γ* = −6.50 °C/km and a threshold of −20 °C. This threshold is used as the typical temperature range of standard onshore wind turbines, which are not specifically adjusted to cold climate, operate from −20 °C to 50 °C^52^.
- Wet snowfall days: This indicator is derived by averaging the model output temperatures at 850 hPa (approx. 1500 ma.s.l.) and 500 hPa (approx. 5500 m.a.s.l.), approximating conditions at 3500 m.a.s.l. Assuming, this height is well above the planetary boundary layer and a lapse rate of *Γ* = −6.50 °C/km, a temperature between −10 °C and −15 °C indicates, that the 0 °C isotherm is located below 2000 m.a.s.l., which is the typical elevation of mountain ridges in Austria. Requiring a daily precipitation of at least 10 mm ensures that enough melting snow is present for cooling the valley atmosphere, enabling wet snow to fall at the valley floors.
- Extreme wind gust speed: This indicator aims to provide insights into widespread wind storms and is aggregated by NUTS3-region. The wind gust speeds are spatially averaged on a daily basis and subsequently the maximum yearly gust speed is determined. Out of these 20 values per NUTS3-region and GWL, the second highest value, corresponding to the 95^th^ percentile is used, an indicator more robust than the maximum.
- Calm days: As typical onshore wind turbines start operating at a wind speed of 2.5 m/s^16^, a calm day is defined to be a day, where the mean daily wind speed in 150m-turbine height is below this threshold. For each pixel, the percentile corresponding to 2.5 m/s in 150 m is determined in the COSMO-REA6 data set^53^, which is reprojected and subsequently mapped to the 10m-wind speed of the models’ GWL-1.0 °C respectively the historical data. After determining the 10m-wind speed corresponding to the determined percentile, the values under the threshold are counted similar to other indicators.

Indicators given in days per year are further calculated in two ways: the annual frequency of the indicators is averaged over the 20 years in a GWL period (indicator version a), and the longest period with consecutive days exceeding the indicator threshold of every year is determined and averaged over 20 years (indicator version b).

### Determining the climate change signal

Indicators for the climate projections are calculated with the same methodology in the corresponding GWL periods. The values of indicator *y* for GWL-X.0 °C are then calculated by adding the climate change signal, that means their difference to the model-specific GWL-1.0 °C, as an average of all models *m* to the observations obs:

2 $${y}_{{GWL}_{X.0}}={y}_{obs}+\sum _{m}({y}_{{GWL}_{X.0,m}}\,-\,{y}_{{GWL}_{1.0,m}})\,.$$

The method of adding the averaged trend of climate models to the historical data set is used as i) the historical data serves as ground truth because it originates in observations^30,31^ and ii) the used bias-adjustment methodology of quantile mapping^41^ does not adjust the persistence of weather patterns. This means that the climate projections, although the values are in a similar range like the historical data, still could suffer from biases connected to spatial or temporal internal variability or persistence. To mitigate that, the model-internal differences to GWL-1.0 °C are calculated and added to the more reliable historical data, assuming that the potential bias is time-independent. Further, a relative approach is not used as it is more sensitive to values close to zero in the GWL-1.0 °C period.

## Data Records

The individual maps for every hazard for the historical observations (corresponding to GWL-1.0 °C), and the future GWLs-2.0 °C, 3.0 °C and 4.0 °C are available publicly on Zenodo^23^. The structure is as follows:

- Maps.zip contains one *.tif file per indicator and GWL, providing the data on a grid-cell basis.
- Tables.zip contains a tabular format for every indicator, where one entry for every of Austria’s NUTS3-regions is shown for every GWL. Tables for maximum, minimum and average NUTS3 values are available.
- Tables_percentages.zip contains similar tables, but displays the changes of the GWLs in relation to the historical value in %. Regions, in which the historical value is zero, are filled with ‘nan’.
- The file indicator_number_and_unit_dicts.txt includes Python-ready dictionaries, linking the indicator numbers and units to the indicator names, which are also used as file names.
- Plots.zip contains one *.png file per indicator, visualising the difference between the values in the historical observations (GWL-1.0 °C, 2001–2020) and the other GWLs, similar to Figs. 2 to 4.

Table 4 shows the units of the individual indicators as well as the assigned number used in file names for better orientation and clarification.

**Table 4:** Indicator numbers, names, and units used for the file names and values in the provided tables and maps.

#	Name	Unit
01a	desert days	days/year
01b	average longest period of desert days	period length in days
02a	days in heat waves	heat wave days/year
02b	average longest period of heat wave days	period length in heat wave days
03a	average maximum temperature	°C
03b	absolute maximum temperature	°C
04a	tropical nights	days/year
04b	average longest period of tropical nights	period length in days
05a	cooling degree days	days/year
05b	average longest period of cooling degree days	period length in days
06	average maximum soil temperature	°C
07	warm soil days	days/year
08a	frost days	days/year
08b	average longest period of frost days	period length in days
09a	ice days	days/year
09b	average longest period of ice days	period length in days
10a	extreme ice days	days/year
10b	average longest period of extreme ice days	period length in days
11	ice throw days	days/year
12	heating degree days	days/year
13	heating degree sum	sum
14	average maximum daily precipitation	mm
15	average maximum three-day precipitation	mm
16	wet snowfall days	days/year
17	snowfall days	days/year
18	fluvial flood days	days/year
19	debris flow days	days/year
20	storm days	days/year
21	strong storm days	days/year
22	extreme wind gust speed	m/s
23a	calm days	days/year
23b	average longest period of calm days	period length in days
24	very high fire danger days	days/year
25	extreme fire danger days	days/year
26	years with one-month drought	number of years
27	years with three-month drought	number of years
28	years with extreme low flow	number of years
29a	humid days	days/year
29b	average longest period of humid days	period length in days
30	thunderstorm days	days/year
31	lightning-induced fire danger days	days/year

## Technical Validation

**Fig. 6:**
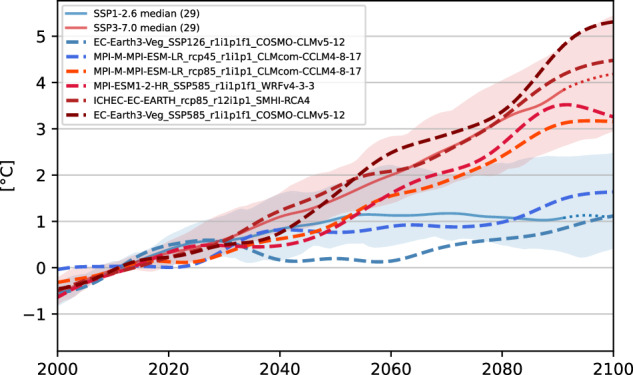
Comparison of the temperature anomaly trend (20-year rolling mean) for the Austrian domain in relation to the 20-year mean of 2001–2020 (GWL-1.0 °C) in °C. The regional climate projections used in this study (dashed) are compared to the SSP1-2.6 (blue) and SSP3-7.0 (red) scenario of the CMIP6 ensemble (29 models). The shaded areas show the 10^th^ and 90^th^ percentile of the corresponding SSP scenario, the solid lines the median.

Figure 6 compares the development of temperature for the climate projections used in this study in relation to the mean of 2001–2020 (GWL-1.0 °C) in the Austrian domain, compared to two Shared Socioeconomic Pathway (SSP) scenarios of the CMIP6 generation^35^. The six selected models (dashed lines) cover a broad spectrum of plausible temperature scenarios, ranging from one model showing less warming than the SSP1-2.6 ensemble mean (solid blue, fulfilling the two-degree target) to one representing the 90^th^ percentile of the SSP3-7.0 ensemble (solid red, business as usual scenario) at the end of the century. The ensemble therefore aims to cover the bandwidth of SSP1-2.6 and SSP3-7.0. As the chosen models therefore cover different emission scenarios, they reach the GWLs in different decades, as Table 3 suggests. However, once reached, the climate within a GWL is similar and nearly independent of the emission scenario^10^.

**Fig. 7:**
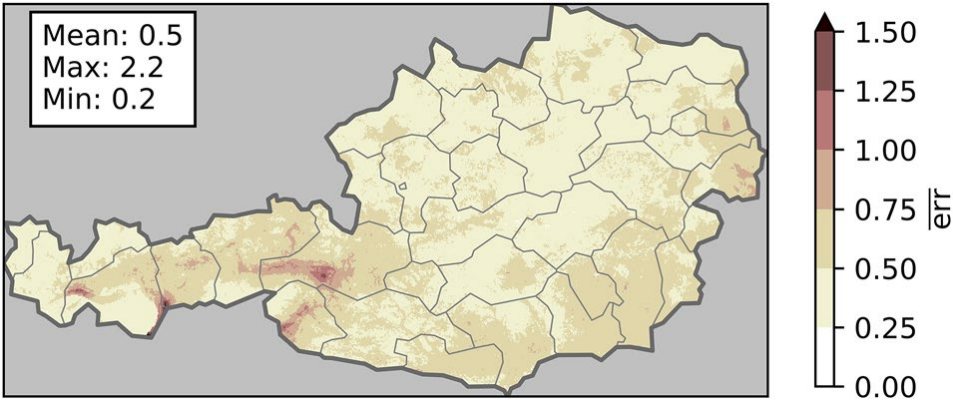
Normalized error $\overline{err}$ in terms of the historical standard deviation, averaged over all 41 indicators.

The historical data sets and advanced indicators were extensively validated against observations in the original publications^29–31,42,43,49,54^, whereas the validation in this study covers the assessment of climate projection downscaling and bias-adjustment^41^. To validate the performance and biases of the climate projections, the differences between the GWL-1.0 °C periods, produced by the average of the climate projections and the actual GWL-1.0 °C period (2001–2020) in the historical data is assessed. For that purpose, the normalized error $err$ for every indicator *y* is calculated on a grid cell level:

3 $$err=\frac{| {y}_{{GWL}_{1.0}}-{y}_{obs}| }{den},$$

where the denominator is

4 $$den=\{\begin{array}{ll}{\sigma }_{obs} & if\,{\sigma }_{obs}\ne 0\\ | {y}_{{GWL}_{1.0}}-{y}_{obs}|  & if\,{\sigma }_{obs}=0\,and\,| {y}_{{GWL}_{1.0}}-{y}_{obs}| \ne 0,\\ \infty  & otherwise\end{array}$$

with *σ*_obs_ being the standard deviation of the 20 yearly values in the historical data (2001–2020). Figure 7 shows the average normalized error $\overline{err}$ for all 41 indicator maps. The climate projections show a spatially averaged error of 0.5*σ*_obs_ for every indicator. The highest values (shaded in red) are observed in Austria’s west, mainly caused by the thunderstorm days indicator, as the climate projection’s lightning-active area is slightly shifted compared to the observations. This is a product of the coarse resolution of the temperatures on pressure levels used to calculate the lightning probability^42^. The fact that no elevation dependency or other geographical dependency is visible, highlights the quality of the data set and the methodology used to bias-adjust and regionalize the data.

**Fig. 8:**
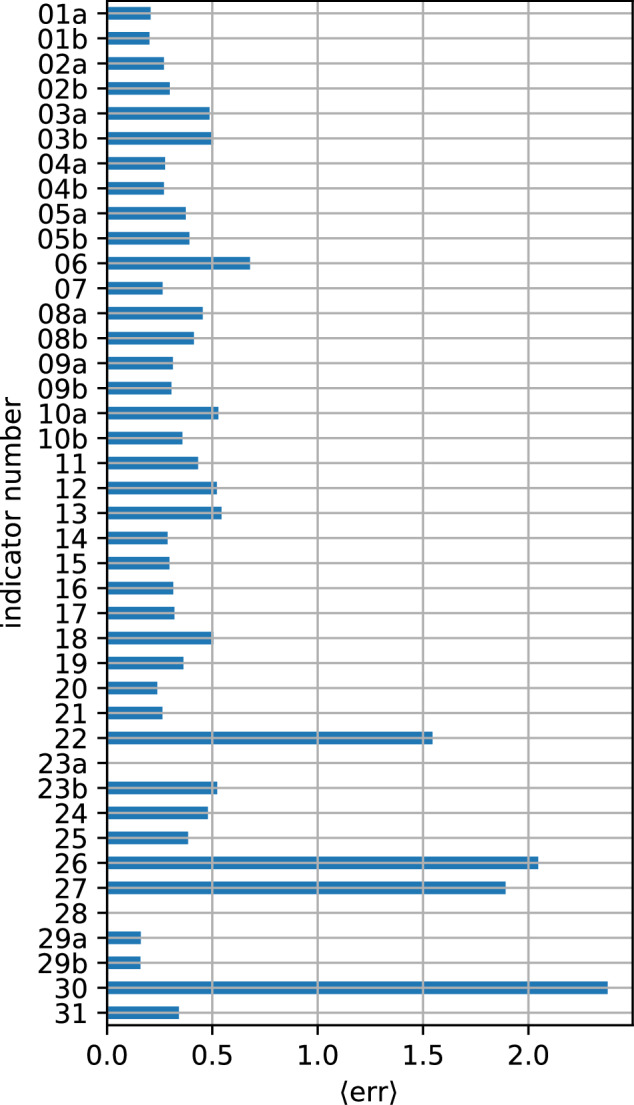
Spatially averaged normalized error ⟨err⟩ in terms of the historical standard deviation for all 41 indicators. The indicator numbers displayed are referenced in Table 4.

Figure 8 shows the spatially averaged normalized error ⟨err⟩ for every of the 41 indicators. It shows that peak over respectively under threshold indicators utilizing bias-adjusted temperature and precipitation data (e.g. indicator 01a - 10b for temperature and 14-16 for precipitation) show the smallest ⟨err⟩. Indicators representing extreme conditions for storm gusts (indicator 22) as well as the aforementioned thunder storm days (indicator 30) are amongst the highest values of ⟨err⟩, which indicates, that the climate projections struggle to reproduce extreme values observed in the historical period. Furthermore, a double penalty applies to the thunderstorm days: storm locations are misplaced toward regions with fewer events, while areas with high occurrence are underestimated. The high ⟨err⟩ values for drought indicators (26 and 27) suggest, that models struggle to reproduce the persistency of drought conditions. The calm days (indicator 23a) by definition do not yield a bias in the GWL-1.0 °C periods, as the calm days are assessed with an percentile approach on an individual model basis (see section 2). However, persistence of calm days (indicator 23b) is not accurately reproduced by the climate projections. No extreme low flow (indicator 28) is observed within GWL-1.0 °C periods, which is why ⟨err⟩ is 0 for that indicator.

**Fig. 9:**
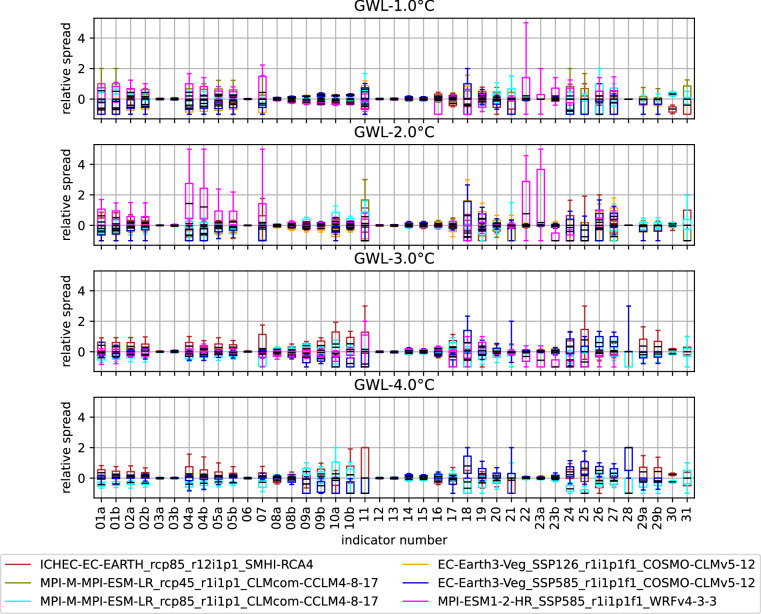
Relative individual model spread boxplots for all 41 indicators. Medians are shown in black. Individual models are color-coded and visible in the legend. Whiskers correspond to the 10^th^ and 90^th^ percentile. The indicator numbers displayed are referenced in Table 4.

Figure 9 analyses the individual model spread of the climate projections. Displayed are boxplots of pixel-wise differences of the individual models contributing to a GWL with respect to the cross-model GWL mean, expressed relative to that mean. The GWL-1.0 °C panel highlights the well-known alpine cold temperature bias for the CMIP5 EURO-CORDEX models^55^ as the CMIP5 model spreads for most temperature indicators (indicators 01-02 and 04-10) are below zero. The maximum temperatures (03 and 06) are not affected due to the percentile-based bias-adjustment. The bias decreases with rising GWLs, suggesting that the climate change signal dominates over model uncertainty at higher warming levels. The model spread for cold indicators (08–13) and especially ice throw days (11) increases with rising GWL as the GWL mean and therefore the denominator decreases with rising temperature. Precipitation and wind based indicators (14–19 respectively 20–23) generally are more noisy than temperature indicators, an effect of internal model variability for precipitation and surface wind parametrisation on smoothed topography for wind. Maximum precipitation (14 and 15) as well show little spread due to bias-adjustment. Fire (24 and 25), drought (26–28) and lightning indicators (30 and 31) generally show high spread, as they combine the variability of multiple meteorological variables including humidity (29), wind speed, radiation, precipitation and temperature on multiple atmospheric levels.

Further, it is notable that the CMIP6 model MPI-ESM1-2-HR_SSP585_r1i1p1f1_WRFv4-3-3 (pink boxes) is a positive the outlier in most temperature indicators as well as in calm day indicators (23), a behaviour, which weakens with increasing GWL and vanishes for GWL-3.0 °C. This is opposed be the behaviour of the CMIP5 model ICHEC-EC-EARTH_rcp85_r12i1p1_SMHI-RCA4 (darkred boxes), which shows rising positive spread with increasing GWL. This highlights that global GWLs can lead to different regional effects, which are dependent on internal variability.

Nevertheless, limitations of the data set remain:

- Climate model ensemble: Due to the high computational expense of downscaling the climate projections to a horizontal resolution suitable for Austria’s complex orography, which is prioritized to accurately represent the climatic conditions in Austria’s highly populated valleys, the number of projections is limited to six models. While the effect of a small ensemble is mitigated by the careful selection of the models, which are covering multiple emission scenarios and climate model generations, and using the GWL approach, which is enabling a larger ensemble for low GWLs, uncertainty regarding the internal variability of the projections remains.
- Missing large-scale flood indicator: Large scale precipitation events, leading to flooding of large rivers like Inn and Danube, cannot be captured due to the geographical limitation of the Austrian territory. Further, our climate projections struggle in producing high enough precipitation on consecutive days that eventually leads to such events, making this indicator not feasible to provide.
- Drought indicators: The SPEI is only calculated on a (three-)monthly basis. As the reference period is only 20 years, this leads to a small sample to calculate the SPEI from, possibly resulting in bad fitting parameters for individual grid cells.
- Assessing extreme low flows: The indicator representing extreme low flow conditions neglects contributions from alpine snow and glacier melt. As the impact of snow melt is most relevant in spring and early summer, the months May and June were excluded from the considered months when assessing this indicator.
- Storm days: The underlying data set for wind gusts suffers from elevation-interpolation problems, resulting in unrealistically high wind gust speeds on mountain tops. Therefore, an artificial limit for storm days and strong storm days were introduced, which are 60 respectively 30 days per year.
- Indicator selection: We believe that there is still improvement in indicator selection to further concentrate on the needs of the energy sector. An estimation of hail would be beneficial for planning of PV locations and mitigating PV efficiency loss^56^. An estimation of water temperature in rivers could inform hydro power and thermoelectric potential^57^. Both phenomena currently lack suitable methodology and statistics as they cannot be derived from climate projection outputs.
- Threshold selection: Some of the hard thresholds could be improved by fine-adjustment and considering more targeted effects on specific components of the energy infrastructure. Heat indicators could be for example produced with multiple thresholds specifically representing a certain vulnerability like efficiency loss in power lines^58^. Suitable thresholds for e.g. cooling and heating degree days are further dependent on the design of buildings and geographical location^59– 61^.
- Multi-hazards and compound events: Due to the limited geographical extent of the study region and scientific basis of compound events in alpine terrain^62^, no compound events besides the combined indicator of lightning and fire risk are presented in ROBINE-AT. Further, many compound extreme events relevant for Austria’s energy sector involve factors beyond pure meteorological causes like fuel prize shocks^14^, which are beyond the scope of this study but subject to further research.

## Usage Notes

The data produced in this study is aimed to be used together with exposure and vulnerability maps to generate risk or impact maps for Austria’s energy system and infrastructure. Similar maps could be derived for other geographical regions with complex orography using the same methodology and the Python script provided at^63^.

## Data Availability

The data is available publicly on Zenodo under 10.5281/ZENODO.14697703^23^.

## Code availability

For reproduce-ability, the script calculating the indicators is publicly available on Zenodo under 10.5281/zenodo.14973624^63^.
